# The Epidemiology of Pathogens in Community‐Acquired Pneumonia Among Children in Southwest China Before, During and After COVID‐19 Non‐pharmaceutical Interventions: A Cross‐Sectional Study

**DOI:** 10.1111/irv.13361

**Published:** 2024-08-15

**Authors:** Ruling Yang, Hongmei Xu, Zhenzhen Zhang, Quanbo Liu, Ruiqiu Zhao, Gaihuan Zheng, Xiaoying Wu

**Affiliations:** ^1^ Department of Infectious Diseases Children's Hospital of Chongqing Medical University Chongqing China; ^2^ National Clinical Research Center for Child Health and Disorders Ministry of Education Key Laboratory of Child Development and Disorders Chongqing China; ^3^ The First Batch of Key Disciplines on Public Health in Chongqing Chongqing Key Laboratory of Child Rare Diseases in Infection and Immunity Chongqing China

**Keywords:** community‐acquired pneumonia, COVID‐19, influenza virus, *Mycoplasma pneumoniae*, paediatric, pathogen epidemiology

## Abstract

**Objective:**

This study aimed to investigate the pathogen epidemiology of community‐acquired pneumonia (CAP) among children in Southwest China before, during and after the COVID‐19 non‐pharmaceutical interventions (NPIs).

**Methods:**

Pathogen data of hospitalised children with CAP, including multiple direct immunofluorescence test for seven viruses, bacterial culture and polymerase chain reaction (PCR) for *Mycoplasma pneumoniae* , were analysed across three phases: Phase I (pre‐NPIs: 1 January 2019 to 31 December 2019), Phase II (NPI period: 1 January 2020 to 31 December 2020) and Phase III (post‐NPIs: 1 January 2023 to 31 December 2023).

**Results:**

A total of 7533 cases were enrolled, including 2444, 1642 and 3447 individuals in Phases I, II and III, respectively. *M. pneumoniae* predominated in Phases I and III (23.4% and 35.5%, respectively). In Phase II, respiratory syncytial virus (RSV) emerged as the primary pathogen (20.3%), whereas detection rates of influenza A virus (Flu A) and *M. pneumoniae* were at a low level (1.8% and 9.6%, respectively). In Phase III, both Flu A (15.8%) and *M. pneumoniae* epidemic rebounded, whereas RSV detection rate returned to Phase I level, and detection rates of *Streptococcus pneumoniae* and *Haemophilus influenzae* decreased significantly compared to those in Phase I. Detection rates of adenovirus and parainfluenza virus type 3 decreased phase by phase. Age‐stratified analysis and monthly variations supported the above findings. Seasonal patterns of multiple pathogens were disrupted during Phases II and III.

**Conclusions:**

COVID‐19 NPIs exhibited a distinct impact on CAP pathogen epidemic among children, with post‐NPIs increases observed in *M. pneumoniae* and Flu A prevalence. Continuous pathogen monitoring is crucial for effective prevention and control of paediatric CAP.

## 1 Introduction

Since the outbreak of coronavirus disease 2019 (COVID‐19) at the end of 2019, it has spread rapidly across multiple countries and regions worldwide [1]. The World Health Organization (WHO) declared it a global public health emergency in January 2020 and a pandemic on 11 March 2020 [2]. As a newly emerging infectious disease, COVID‐19 has profoundly impacted the healthcare systems and human societies [3]. To control the spread of COVID‐19, governments implemented large‐scale non‐pharmaceutical interventions (NPIs), including social distancing, wearing masks, community lockdowns and so on. Although these measures significantly reduced the transmission of the novel coronavirus, they also influenced the epidemiological characteristics and pathogen spectra of respiratory tract infections. The overall incidence of respiratory viral diseases decreased by 23%–94% during the COVID‐19 pandemic from 2020 to 2021, with viral detection rates declining by 0%–98% [4]. A deeper understanding of the impact of COVID‐19 NPIs on non‐COVID respiratory diseases can help elucidate the impact of human–environment interactions on respiratory diseases and the relationships between various pathogens. This understanding can provide essential insights into the prevention and treatment of respiratory diseases during the post‐NPIs era.

Over the past 4 years, China has undergone a series of adjustments and evolutions in the context of COVID‐19 prevention and control strategies [5]. On 23 January 2020, Wuhan was placed under lockdown, signifying China's substantial and decisive measures to curb the comprehensive spread of COVID‐19 pandemic. The NPIs in 2020 in China were the most sustained and stringent. As time progressed, China's strategies evolved into a dynamic ‘zero‐COVID’ policy since 2021, which aimed at balancing pandemic control with society's normal functioning efficiently and under which regional lockdown and relaxation were constantly alternating. Finally, on 8 January 2023, China officially implemented the adjustment of COVID‐19's infectious disease prevention and control level from Class A to Class B. This major policy adjustment was considered the conclusion of the dynamic ‘zero‐COVID’ policy, lifting NPIs including mandatory SARS‐CoV‐2 testing and marking China's formal entry into a new phase of reopening.

Following the complete relaxation of NPIs in China, the epidemiology of pathogens causing community‐acquired pneumonia (CAP) in children remains unclear Therefore, further related research is needed to provide evidence for development of the scientifically grounded strategies to prevent and treat CAP in children, thereby further reducing its incidence and mortality. Hence, this study aims to analyse the pathogen surveillance data of children hospitalised with CAP to explore the evolution of pathogens before, during and after the COVID‐19 NPIs.

## 2 Materials and Methods

### 2.1 Study Design and Participants

This study involved analysing hospitalised children diagnosed with CAP at the Children's Hospital of Chongqing Medical University, Southwest China's largest tertiary paediatric hospital. The study was conducted in three phases: Phase I from 1 January 2019 to 31 December 2019, referred to as the pre‐NPI period; Phase II from 1 January 2020 to 1 December 2020 (we selected the data in 2020 as representative for researching the impact of implementing NPIs on the pathogen epidemiology), referred to as the NPI period; and Phase III from 1 January 2023 to 31 December 2023, referred to as the post‐NPI period. Children less than 18 years of age who met the diagnostic criteria for CAP outlined in the ‘Guidelines for Diagnosis and Treatment of Community‐Acquired Pneumonia in Children (2019 Edition)’ [6] were included in this study. Individuals who met any of the following criteria were excluded from the study: (1) failure to complete all respiratory pathogen tests required in this study; (2) hospitalisation diagnosis of tuberculosis, foreign body aspiration, or drowning; and (3) congenital airway abnormalities or underlying diseases associated with immunodeficiency.

The study population was divided into the following four age groups: age group 1 (< 1 year), age group 2 (1–3 years), age group 3 (3–6 years) and age group 4 (6–18 years).

### 2.2 Specimen Collection and Detection

According to the clinical requirements of the patients, respiratory specimens were collected upon admission for microbiological testing, including deep sputum, nasopharyngeal aspirates or bronchoalveolar lavage obtained via the standard collection method. Patients that were included in the study were required to complete the following three tests. First, multiple direct immunofluorescence assay kit (Diagnostic Hybrids, Athens, OH, USA) was used to simultaneously detect seven common viral pathogens, including respiratory syncytial virus (RSV), adenovirus (ADV), influenza A virus (Flu A), influenza B virus (Flu B) and parainfluenza viruses 1–3 (PIV1, PIV2 and PIV3) [7]. Second, specimens were sent immediately for culture using blood agar (Antu Bioengineering Co., Ltd., Zhengzhou, China), and bacterial identification was performed using the fully automated bacterial identification and drug sensitivity analysis system (MB50, Becton, Dickinson and Company). Third, a real‐time fluorescence quantitative polymerase chain reaction (PCR) detection kit (SLAN‐96P, Hongshi Medical Technology Co., Ltd, Shanghai, China) was used to detect *Mycoplasma pneumoniae* . All the tests were performed by experienced professionals in accordance with the operating procedures and instructions mentioned on the reagent kits [8]. A mixed infection was considered if two or more pathogens were detected.

### 2.3 Data Collection

The following data of the patients were collected from the electronic medical records of the hospital: ID number, sex, age, province of residence, clinical diagnosis, date of sampling, sample type and pathogen detection results. To protect patient privacy, data collection and analysis did not include personal identity information such as names, residential addresses and phone numbers. If a child was readmitted for CAP within less than 1 week, the data collected during the initial hospitalisation were included in the analysis. We calculated the detection rate as follows: the detection rate = number of samples with pathogens detected/total number of samples tested.

### 2.4 Ethical Considerations

The study protocol was approved by the Ethics Committee of the Children's Hospital of Chongqing Medical University. The study was conducted in accordance with the tenets of Declaration of Helsinki. Because the three pathogen tests were routinely performed during the clinical treatment of children and the data did not involve personally identifiable information, the requirement for informed consent was waived.

### 2.5 Statistical Analysis

Statistical analyses were conducted using IBM SPSS software (Version 25.0; IBM, Chicago, IL, USA) to compare the differences in pathogen detection rates and case numbers. Categorical variables were presented as counts and percentages. Two‐tailed chi‐square, corrected chi‐square and Fisher's exact tests were performed as required. A two‐sided *p* value less than 0.05 was considered statistically significant.

## 3 Results

### 3.1 Study Population

A total of 7533 children with CAP formed the study population, including 2444, 1642 and 3447 individuals in Phases I, II and III, respectively (Table 1). Notably, the number of patients in Phase III increased by 41.0% and 109.7% compared to Phases I and II, respectively. The proportion of children aged < 1 year was the highest in Phase II (52.7%), which was significantly higher than that in Phases I and III (χ^2^ values were 56.331 and 344.056, with *p* values < 0.001 for both). The proportion of children aged 3–6 years in Phase III was significantly higher than that in Phases I and II (χ^2^ values were 15.712 and 109.288, with *p* values < 0.001 for both). Moreover, the proportion of children aged > 6 years was also significantly higher in Phase III than in Phases I and II (χ^2^ values were 224.565 and 253.430, with *p* values < 0.001 for both).

**TABLE 1:** Comparison of demographic features and pathogen detection rates in the three phases.

	Total	Phase I	Phase II	Phase III	H/χ2	*p*
	*n* = 7533	*n* = 2444	*n* = 1642	*n* = 3447		
Variables						
Sex (male)	4434 (58.9)	1473 (60.3)	1023 (62.3)	1938 (56.2)	19.941	< 0.001
Age						
< 1 years	2764 (36.7)	996 (40.8)	865 (52.7)	903 (26.2)	361.488	< 0.001
1–3 years	1891 (25.1)	676 (27.7)	452 (27.5)	763 (22.1)	29.778	< 0.001
3–6 years	1640 (21.8)	527 (21.5)	215 (13.1)	898 (26.1)	109.741	< 0.001
> 6 years	1238 (16.4)	245 (10.0)	110 (6.7)	883 (25.6)	398.045	< 0.001
Positive detection sample (rate) by age						
< 1 year	1929 (69.8)	758 (76.1)	538 (62.2)	633 (70.1)	42.534	< 0.001
1–3 years	1403 (74.2)	523 (77.4)	306 (67.7)	574 (75.2)	13.940	0.001
3–6 years	1367 (83.4)	451 (85.6)	160 (74.4)	756 (84.2)	14.701	0.001
> 6 years	1028 (83.0)	200 (81.6)	81 (73.6)	747 (84.6)	8.772	0.012
Positive detection sample (rate) of pathogens						
Overall pathogen detection	5727 (76.0)	1932 (79.1)	1085 (66.1)	2710 (78.6)	114.138	< 0.001
Single pathogen detection	3968 (52.7)	1303 (53.3)	798 (48.6)	1867 (54.2)	14.404	0.001
Mixed pathogen detection	1759 (23.4)	629 (25.7)	287 (17.5)	843 (24.5)	41.759	< 0.001

### 3.2 Positivity Rates of Respiratory Pathogens in the Three Phases

**FIGURE 1:**
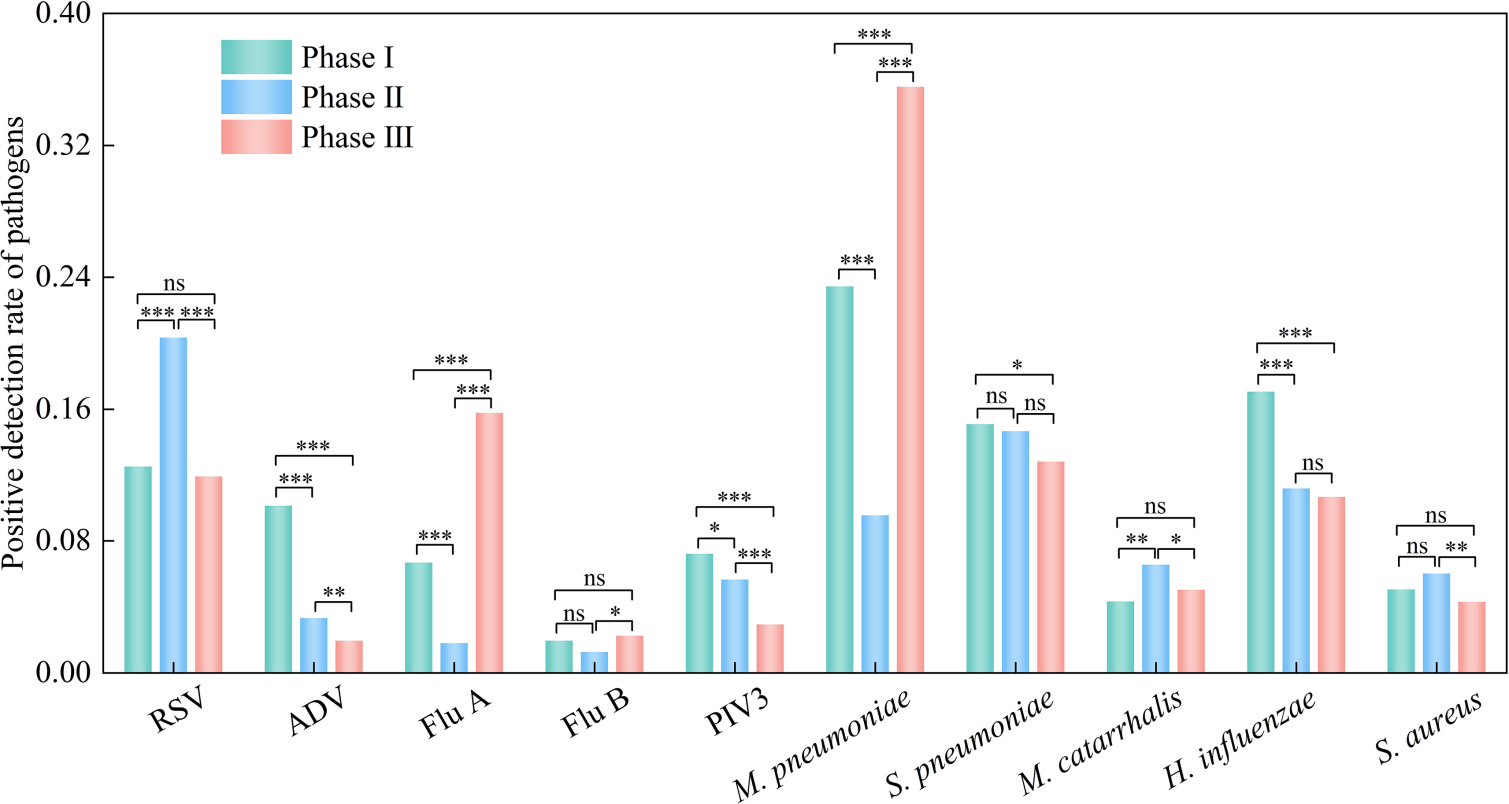
The top 10 respiratory pathogens in detection rate among the three phases. *Note:* *indicates significant difference between two phases. *p < 0.05; **p < 0.01; ***p < 0.001. Abbreviations: ADV, adenovirus; Flu A, influenza A virus; Flu B, influenza B virus; *H. influenzae* , *Haemophilus influenzae* ; *M. catarrhalis* , *Moraxella catarrhalis* ; *M. pneumoniae* , *Mycoplasma pneumoniae* ; PIV3, parainfluenza virus 3; RSV, respiratory syncytial virus; *S. aureus* , *Staphylococcus aureus* ; *S. pneumoniae* , *Streptococcus pneumoniae* .

The overall pathogen detection rate in Phase II was significantly lower than those in Phases I and III (χ^2^ values were 85.563 and 92.252, with *p* values < 0.001 for both). The top five most commonly detected pathogens in Phase I were *M. pneumoniae* (573, 23.4%), *Haemophilus influenzae* (417, 17.1%), *Streptococcus pneumoniae* (369, 15.1%), RSV (306, 12.5%) and ADV (248, 10.1%). In Phase II, the top five most commonly detected pathogens were RSV (334, 20.3%), *S. pneumoniae* (241, 14.7%), *H. influenzae* (184, 11.2%), *M. pneumoniae* (157, 9.6%) and *Moraxella catarrhalis* (108, 6.6%). In Phase III, *M. pneumoniae* (1225, 35.5%) had the highest detection rate, followed by Flu A (544, 15.8%), *S. pneumoniae* (442, 12.8%), RSV (411, 11.9%) and *H. influenzae* (368, 10.7%). Four of the top five detected pathogens were identical across all three phases ( *M. pneumoniae* , *S. pneumoniae* , *H. influenzae* and RSV) (Figure 1).

The detection rate of Flu A in Phase III was significantly higher than that in Phase I (164/2444, 6.7%, ranking seventh) and Phase II (30/1642, 1.8%, ranking 11th), with χ^2^ values of 111.296 and 216.433, respectively, and *p* values of < 0.001 for both. The detection rate of *M. pneumoniae* in Phase III was also significantly higher than that in Phases I and II, with χ^2^ values of 98.620 and 379.387, respectively, and *p* values of < 0.001 for both. Compared to Phase II, the detection rate of *M. pneumoniae* in Phase III increased by 26.0%, and compared to Phase I, it increased by 12.1% (Figure 1).

In Phase II, RSV unexpectedly emerged as the predominant pathogen, with a significantly higher detection rate compared to Phases I and III (χ^2^ values were 45.473 and 63.064; *p* values were < 0.001 for both). ADV and PIV3 exhibited significant decreasing trends across the three phases. The detection rate of ADV ranked fifth in Phase I (248, 10.2%), dropped to eighth in Phase II (55, 3.3%) and further decreased to tenth in Phase III (68, 2.0%). The detection rate of PIV3 in Phase III (102/3447, 3.0%, ranking eighth) decreased by 4.3% and 2.7% compared to that in Phase I (177/2444, ranking sixth) and Phase II (93/1642, ranking seventh), respectively (Figure 1).

Despite the relaxation of NPIs in Phase III, the detection rates of *S. pneumoniae* and *H. influenzae* continued to decline, with rates of 12.8% and 10.7%, respectively. These rates were significantly lower than those in Phases I (15.1% and 17.1%, respectively) (χ^2^ = 6.237, *p* = 0.013; χ^2^ = 50.498, *p* < 0.001) (Figure 1).

### 3.3 Prevalence of Respiratory Pathogens in the Three Phases by Age Groups

**FIGURE 2:**
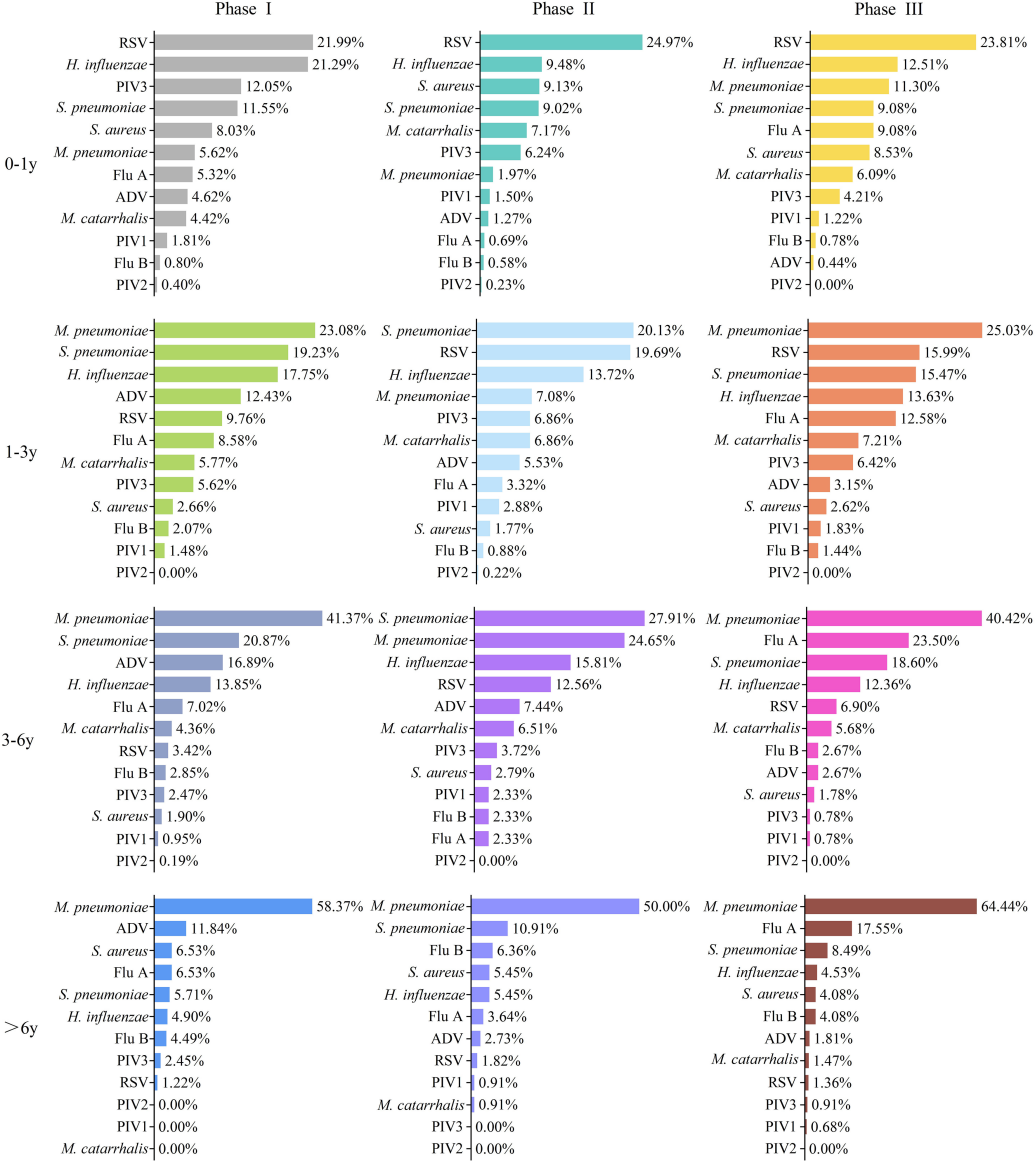
Detection rates of respiratory pathogens in the three phases by age groups. Abbreviations: ADV, adenovirus; Flu A, influenza A virus; Flu B, influenza B virus; *H. influenzae* , *Haemophilus influenzae* ; *M. catarrhalis* , *Moraxella catarrhalis* ; *M. pneumoniae* , *Mycoplasma pneumoniae* ; PIV1, parainfluenza virus 1; PIV2, parainfluenza virus 2; PIV3, parainfluenza virus 3; RSV, respiratory syncytial virus; *S. aureus* , *Staphylococcus aureus* ; *S. pneumoniae* , *Streptococcus pneumoniae* .

The pathogen detection rates in Phases I and III were significantly higher than those in Phase II across all age groups (except for age group 4, where Phase I was higher than Phase II but without statistical significance) (Table 1). The top five most commonly detected pathogens in each phase of age group 1 was RSV, followed by *H. influenzae* and *S. pneumoniae* . In age group 2, those were *M. pneumoniae* , RSV, *S. pneumoniae* and *H. influenzae* . In age group 3, those were *M. pneumoniae* , *S. pneumoniae* and *H. influenzae* . In age group 4, those were *M. pneumoniae* and *Staphylococcus aureus* , and *M. pneumoniae* was the first most commonly detected pathogen in each phase, with a detection rate exceeding 50% (Figure 2).

Age‐stratified pathogen analysis (Figure 2) showed that the detection rate trends of *M. pneumoniae* , Flu A, RSV and ADV were almost consistent with the trends in all cases. In each age group, the detection rate of *M. pneumoniae* in Phase III was significantly higher than that in Phase II (χ^2^ values of 61.265, 61.052, 18.436 and 8.734; *p* values were < 0.001, < 0.001, < 0.001 and 0.003, respectively) and comparable to that of Phase I. The detection rates of Flu A in Phase II were significantly lower than those in Phases I and III (only in age group 4, between Phases II and I, the detection rates showed no statistical difference, with χ^2^ = 1.196, *p* = 0.274). In Phase II, Flu A cases were sporadic in all age groups, whereas in Phase III, the detection rates were significantly higher than that in Phase I (with χ^2^ values of 10.137, 6.007, 62.714 and 18.119 and corresponding *p* values of 0.001, 0.014, < 0.001 and < 0.001). The detection rates of RSV were highest in Phase II (significant differences existed between Phases I and II in age group 2, between Phases I and II in age group 3 and between Phases II and III in age group 3, with χ^2^ values of 22.521, 22.405 and 7.537, *p* values of < 0.001, < 0.001 and 0.006, respectively). ADV detection rates decreased over phases, with only no significant differences between Phases II and III in age groups 1, 2 and 4 (χ^2^ values of 3.607, 4.174 and 0.085; *p* values of 0.058, 0.041 and 0.770, respectively).

*S. pneumoniae* , *H. influenzae* , *S. aureus* and *M. catarrhalis* detection rates in all four age groups showed no significant differences among the three phases. However, in age group 1, the detection rate of *H. influenzae* in Phase III (113/903, 12.5%) was significantly lower than that in Phase I (212/996, 21.3%) (χ^2^ = 25.687, *p* < 0.001).

### 3.4 Monthly Patterns of the Common Respiratory Pathogens in the Three Phases

**FIGURE 3:**
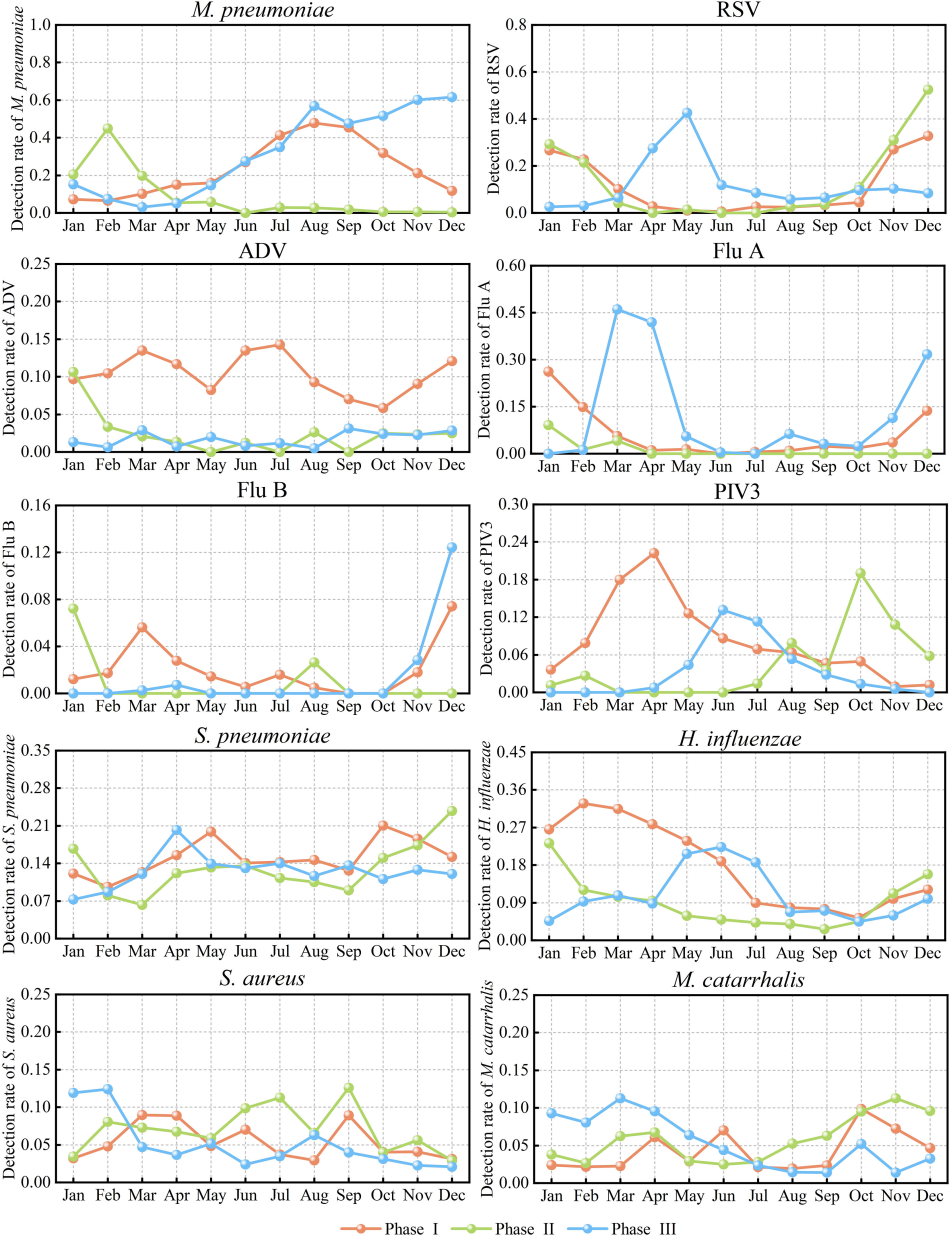
Variations in monthly detection rates of the top 10 respiratory pathogens in the three phases. Abbreviations: ADV, adenovirus; Flu A, influenza A virus; Flu B, influenza B virus; *H. influenzae* , *Haemophilus influenzae* ; *M. catarrhalis* , *Moraxella catarrhalis* ; *M. pneumoniae* , *Mycoplasma pneumoniae* ; PIV3, parainfluenza virus 3; RSV, respiratory syncytial virus; *S. aureus* , *Staphylococcus aureus* ; *S. pneumoniae* , *Streptococcus pneumoniae* .

The epidemiology of the 10 most commonly detected pathogens over the months in the three phases were analysed (Figure 3). In Phase I, it was found that Flu A mainly circulated in January, February and December, with January being the peak of the epidemic (26.2%, 65/248). Moreover, the epidemic was lowest between March and November, with a detection rate of < 6%. In Phase II, the prevalence of Flu A almost stagnated with no peak epidemic period, and no cases of influenza A were detected from April to December. However, in Phase III, Flu A reached its annual peak in March (46.1%, 176/382) and showed a small peak in December (31.7%, 166/523), with its detection rate significantly surpassing that of December in Phase I (χ^2^ = 29.308, *p* < 0.001). Furthermore, it was observed that Flu A co‐circulated with Flu B.

In Phase I, *M. pneumoniae* was detected throughout the year, peaking in August (47.8%, 98/205). However, in Phase II, the epidemic pattern of *M. pneumoniae* was markedly different from that during Phase I, with almost no cases detected from June to December. In Phase III, from January to September, the prevalence of *M. pneumoniae* mirrored that in Phase I, peaking in August (56.8%, 117/206). However, unlike in Phase I, the epidemic did not gradually decrease from October to December but remained high, reaching an annual peak in December (61.6%, 322/523), which was higher than that in Phase I.

The epidemics of RSV in Phases I and II were broadly consistent, prevalent in November, December, January and February, peaking in December (32.8%, 84/256; 52.5%, 126/240, respectively), and nearly absent from March to October. However, in Phase III, the seasonal RSV pattern shifted to April to June, with the annual peak occurring in May (42.6%, 107/251).

In Phase I, ADV was detected throughout the year, with monthly detection rates ranging between 5.8% and 14.3%. However, in Phase III and from February to December in Phase II, sporadic or no detection was observed each month (< 4.0%). In Phase II, PIV3 was rarely detected from January to July; however, the detection rates increased from August, peaking in October (19.0%). The detection rates of PIV3 from October to December were significantly higher than those in the same months of Phase I (χ^2^ values of 20.372, 19.555, 8.132; *p* values of < 0.001, < 0.001 and 0.004, respectively) and Phase III (χ^2^ values of 46.719, 32.838 and 27.924; all *p* values of < 0.001). The peak epidemic month of PIV3 in Phase III was observed in June (13.2%, 33/251), which was slightly later than that in Phase I (April, 22.2%, 40/180). Flu B was sporadically detected in most months of Phase I, with almost no cases detected in Phases II and III. However, in December of Phase III, the detection rate increased to 12.4% (65/523), which was the highest among the three phases.

*S. pneumoniae* , *S. aureus* , *M. catarrhalis* and *H. influenzae* were detected monthly. No apparent epidemic season was evident for *S. pneumoniae* , *S. aureus* or *M. catarrhalis* . Additionally, there were no significant monthly differences were in the detection rates of *S. pneumoniae* , *S. aureus* or *M. catarrhalis* across the three phases. In Phase I, *H. influenzae* exhibited relatively higher monthly detection rates from January to June. During the first 6 months of Phase II, *H. influenzae* detection rates were significantly lower than in the same months of Phase I. In Phase III, *H. influenzae* detection rates from May to July remained relatively high throughout the year.

### 3.5 Co‐infections Caused by Pathogens

The most common pathogen combinations were *S. pneumoniae* with *M. pneumoniae* and *S. pneumoniae* with RSV, with 201 (11.0%) and 175 (9.6%) cases, respectively. Additionally, it was found that Flu A and *M. pneumoniae* easily co‐infected with other pathogens. For example, there were 123 cases (6.8%) of Flu A + *M. pneumoniae* , 82 (4.5%) of Flu A + *S. pneumoniae* , 89 (4.9%) of Flu A + *H. influenzae* , 103 (5.7%) of *M. pneumoniae* + *H. influenzae* and 84 (4.6%) of *M. pneumoniae* + *S. aureus* .

## 4 Discussion

This study revealed the pathogenic epidemiological characteristics and pathogen spectrum evolution of childhood CAP in Southwest China before, during and after the COVID‐19 NPIs. Furthermore, this study explored the potential reasons for these evolutions. It was found that the epidemic and seasonality of specific pathogens were interrupted in Phase II. In particular, the epidemics of *M. pneumoniae* , Flu A and ADV were significantly suppressed. However, the prevalence of Flu A and *M. pneumoniae* rebounded during the post‐NPIs period. Both *M. pneumoniae* and Flu A played a significant role in the substantial increase in CAP cases in Southwest China in 2023.

The detection rate of *M. pneumoniae* ranked first during Phase I, dropped to fourth during Phase II and then surged back to first during Phase III with an increased detection rate comparing Phase I across three age groups. Traditionally, the detection rate of *M. pneumoniae* tends to rise in late summer and autumn and fall in late winter and spring. However, from August to December 2023, the monthly detection rates of *M. pneumoniae* remained consistently above 40%. The above data indicated that *M. pneumoniae* exhibited a higher prevalence than other respiratory pathogens and a longer epidemic time than before. Furthermore, co‐infection of *M. pneumoniae* with Flu A or *S. pneumoniae* was commonly observed. Similarly, an increase in *M. pneumoniae* prevalence was reported in the autumn and winter of 2023 in Northern China, Netherlands and United States [9, 10, 11].The exact reasons for the increase in *M. pneumoniae* prevalence in 2023 remains unclear; however, it may be related to the inherent cyclical patterns and potential ‘immune debt’ of *M. pneumoniae* . *M. pneumoniae* outbreaks typically occur every 3–5 years. The most recent *M. pneumoniae* outbreak occurred during the end of 2019 and the beginning of 2020 [12]. Given the current outbreak interval of 3–4 years, the increase in *M. pneumoniae* cases in 2023 may align with its regular cyclic pattern. The COVID‐19 NPIs may limit children's exposure to common pathogens and then block the stimulation of the immune system by these pathogens, thereby hindering the individual's establishment of protective immunity against them [13, 14, 15]. The immune gap, which means ‘immune debt’, leads to the continuous accumulation of susceptible individuals [13, 14, 15]. After the lifting of NPIs, a large number of susceptible individuals may easily be infected by the pathogens they come into contact with. Additionally, the increasing resistance of *M. pneumoniae* towards macrolides played an additional role in the heightened prevalence of *M. pneumoniae* in China in 2023 [16].

Following the implementation of NPIs, the global influenza virus activity decreased significantly [17]. Upon cancellation of NPIs, a global rebound of influenza occurred, which surpassed the levels of previous years, with Flu A predominating. The WHO influenza monitoring data revealed a notable peak in global influenza cases from October 2022 to February 2023, reaching the highest level in nearly a decade. Europe experienced its peak in November 2022, the United States in December 2022 and Northern China in February 2023. Flu A detection rates in the present study were 6.7% in 2019, 1.8% in 2020 and 15.8% in 2023. The peak of influenza season in 2023 in this study occurred in March with a detection rate of 46.1%, surpassing the peak of 36.2% in 2019. The findings of this study were consistent with the global influenza trends. The heightened influenza activity beyond pre‐NPI levels may be associated with lifting NPIs, ‘immune debt’ and mechanisms of viral interference. In China, influenza peaked later compared to other countries, possibly due to the strict and prolonged implementation of NPIs. Similar to other countries, there was a lag in the increase in influenza incidence following the removal of NPIs, possibly due to ‘viral interference’ between the novel coronavirus and influenza viruses [18]. During the initial time of NPIs relaxation, the prevalence of novel coronavirus remained relatively high, whereas that of influenza remained low. Subsequently, with the decrease in novel coronavirus circulation, the peak of influenza A was observed in March 2023. However, the seasonality of influenza in Southwest China in 2023 remained essentially unchanged, with epidemics predominantly occurring in winter and spring.

A noteworthy finding of this study was that despite the weakening trend of most viruses during Phase II, RSV increased and became the most detected pathogen (20.3%). This was consistent with the findings of Ye et al. [7] This increase may be related to the mutual interference between RSV and influenza viruses [19]. Additionally, RSV primarily infects infants and toddlers aged less than 3 years, who may be unwilling to wear masks or wear improperly sized masks, thus failing to provide effective respiratory protection [20]. With the lifting of NPIs, the prevalence of RSV was found to return to pre‐NPI levels but exhibited off‐season prevalence. Historically, the prevalence of RSV in Southwest China typically lasts from October to March of the following year [21]. In this study, the epidemic of RSV in Phases I and II followed the seasonal pattern before the commencement of the COVID‐19 pandemic. However, in Phase III, the seasonal peak of RSV shifted to April to June. Other countries also experienced off‐season RSV prevalence in the first year after lifting NPIs [22]. This phenomenon may be related to the prevalence interference between different viruses [23]. Furthermore, infants have lower levels of RSV IgG antibodies, which may also contribute to the early spread of RSV. Currently, no RSV vaccine is available, and infants under the age of 6 months usually acquire RSV immune protection through maternal antibodies transmitted during pregnancy. However, during the COVID‐19 pandemic, pregnant women adhering to NPIs had reduced RSV exposure, lowering the protective antibody generation. This significantly affected infants' immune protection via maternal transmission [24]. Thus, with the lifting of NPIs weakening the RSV epidemic restrictions, susceptible infants and toddlers were infected, thereby promoting the early epidemic of RSV.

Among the four most common bacterial pathogens observed in this study, vaccines for only *S. pneumoniae* and *H. influenzae* are available currently. Following the lifting of NPIs, the prevalence of *S. pneumoniae* and *H. influenzae* significantly decreased compared to the pre‐NPI levels. However, the detection rates of *M. catarrhalis* and *S. aureus* did not change significantly. This phenomenon may be due to increased vaccination awareness during the COVID‐19 pandemic in China, resulting in higher vaccination rates and better immune protection against *S. pneumoniae* and *H. influenzae* [25].

In this study, an increase in the proportion of preschool and school‐age children with CAP was observed in Phase III compared to Phases II and I. This finding is consistent with those of other studies [26]. Flu A and *M. pneumoniae* were mainly observed in older children [27, 28]. The significant increases in the prevalence of *M. pneumoniae* and Flu A in 2023 in the present study may be a crucial factor contributing to changes in age distribution.

However, this study had a few limitations to consider. First, it was observational, potentially susceptible to selection bias. Second, this single‐centre study focused primarily on hospitalised children with CAP, which may have limited the generalisability and external validity of the results. Third, we cannot distinguish specimens between deep sputum and nasopharyngeal aspirates, as they were uniformly labelled as sputum in the test report forms in our hospital. Despite this limitation, the fact that the majority of specimens were nasopharyngeal aspirates, particularly among younger children, ensures that our test results still maintain a good level of comparability. Finally, the detection of pathogens does not necessarily equate to infections. Therefore, a causal link between pathogens and CAP was not established definitively.

## 5 Conclusions

This study shows that COVID‐19 NPIs had varying impacts on epidemics of different respiratory pathogens, highlighting the need for careful consideration of multiple factors when predicting pathogen epidemiological trends. In the first year following the cancellation of NPIs, China experienced a co‐prevalence of influenza and *M. pneumoniae* . In the post‐NPI era, it is essential to establish a ‘new balance’ among known respiratory pathogens, and when the next ‘new coronavirus’ will emerge remains unknown. To ensure preparedness and swift responses to public health challenges, continued pathogen surveillance is essential in the future, particularly in large‐scale multicentre studies. Furthermore, strengthening international cooperation and information sharing is crucial. These efforts will contribute to a comprehensive and in‐depth understanding of respiratory pathogen epidemiology and their interactions, providing theoretical support for precise and effective prevention and control of children's CAP. In addition, proactive strategies like accelerating vaccine development or improvement, increasing vaccine coverage rates, enhancing CAP diagnosis and treatment capacity and promoting health education and literacy should also be pursued.

## Author Contributions

**Ruling Yang:** conceptualisation (lead), methodology (equal), formal analysis (supporting), project administration (equal), writing – original draft (lead), writing – review and editing (equal). **Hongmei Xu:** project administration (equal), data curation (equal), writing – review and editing (equal). **Zhenzhen Zhang:** data curation (equal), investigation (equal), writing – review and editing (equal). **Quanbo Liu:** investigation (equal), supervision (equal), writing – review and editing (equal). **Ruiqiu Zhao:** supervision (equal), visualisation (equal), writing – review and editing (equal). **Gaihuan Zheng:** visualisation (equal), writing – review and editing (equal). **Xiaoying Wu:** conceptualisation (supporting), methodology (equal), formal analysis (lead), writing – original draft (supporting), writing – review and editing (equal).

## Ethics Statement

The study was approved by the ethics committee of Children’s Hospital of Chongqing Medical University [(2024) Annual Luncheon Review (Research) No. 118].

## Consent

In this study, because the three pathogen tests were routinely performed during the clinical treatment of children and the data did not involve personally identifiable information, the requirement for informed consent was waived.

## Conflicts of Interest

The authors declare no conflicts of interest.

## Data Availability

The data that support the findings of this study are available from the corresponding author upon reasonable request.
